# Excessive use of electronic devices among children and adolescents is associated with musculoskeletal symptoms, visual symptoms, psychosocial health, and quality of life: a cross-sectional study

**DOI:** 10.3389/fpubh.2023.1178769

**Published:** 2023-06-29

**Authors:** Sharon M. H. Tsang, Gladys L. Y. Cheing, Andrew K. C. Lam, Andrew M. H. Siu, Peter C. K. Pang, Kin-Chung Yip, Jess W. K. Chan, Mark P. Jensen

**Affiliations:** ^1^Department of Rehabilitation Sciences, The Hong Kong Polytechnic University, Kowloon, Hong Kong SAR, China; ^2^School of Optometry, The Hong Kong Polytechnic University, Kowloon, Hong Kong SAR, China; ^3^Research Centre for SHARP Vision, The Hong Kong Polytechnic University, Kowloon, Hong Kong SAR, China; ^4^Hong Kong Centre for Eye and Vision Research, Hong Kong, Hong Kong SAR, China; ^5^Department of Health Sciences, Brunel University London, Uxbridge, United Kingdom; ^6^Hong Kong Evangelical Church Social Service Limited, Hong Kong, Hong Kong SAR, China; ^7^Department of Rehabilitation Medicine, University of Washington, Seattle, WA, United States

**Keywords:** children and adolescents, electronic device, musculoskeletal symptoms, visual symptoms, psychosocial health, quality of life

## Abstract

**Objective:**

Electronic devices have become an indispensable part of our daily lives. The frequency and duration of device use in children and adolescents have increased drastically over the years and the study of its negative musculoskeletal, visual and psychosocial health impacts is necessary.

**Materials and methods:**

This cross-sectional study aimed to evaluate the associations between electronic device use and the prevalence and severity of musculoskeletal symptoms, visual symptoms, psychosocial health, and quality of life in children and adolescents studying at primary and secondary schools. Data were collected through confidential online and paper-and-pencil questionnaires. Primary 4–5 and Secondary 1–4 students were recruited from 3 schools in Hong Kong. Demographics, frequency and duration of electronic device use, frequencies of musculoskeletal symptoms, visual symptoms, psychosocial health, and quality of life outcomes were measured.

**Results:**

1,058 children and adolescents aged 9–17  years participated. Sixty-one percent and 78% of all students spent more than 2  h per day using electronic devices during school days and weekend/holidays, respectively. Extended electronic device use was associated with increased prevalence and severity of musculoskeletal symptoms (ρ’s = 0.28–0.33, P’s < 0.001), visual symptoms (ρ’s = 0.33–0.35, P’s < 0.001), and poorer device use-related psychosocial health (ρ’s = 0.38–0.47, P’s < 0.001). Secondary school students reported greater device use and severity of symptoms than primary school students.

**Conclusion:**

Excessive electronic device use was associated with increased prevalence and severity of physical and psychosocial symptoms, and such use is more prevalent in adolescents when compared to the children. The findings have important health implications for children and adolescents, suggesting that early intervention is needed to reduce the risk of developing device use-related disorders.

## Introduction

Many youths use electronic devices daily. Over 95% of adolescents aged 13–16 years owned their smartphones in 2018 globally and this figure has increased by 22% since 2014 (1). Electronic devices include smartphones, tablets, computers, and game consoles (2). These devices are used for social networking, studying, and entertainment. Most recently, perhaps due to the need to limit face-to-face activities because of Covid-19, the primary reason to use electronic devices has been shifting from entertainment to education, especially in economically privileged countries where e-learning has been incorporated into the school policy (3). As a result, the frequency and duration of electronic device use in adolescents has increased dramatically (4).

A growing body of research has also been showing that the extended electronic device use is associated with numerous musculoskeletal symptoms including neck/shoulder pain, lower back pain, and arm discomfort (2, 3, 5–9). These symptoms are known to be associated with reduced physical activity, increased medication use, and school absence in adolescents (10–12).

Associations between the use of electronic devices and visual problems have also been reported (2, 3, 7, 13). Prolonged and frequent use of visual display units can lead to the development of Computer Vision Syndrome (CVS), which is associated with a set of specific symptoms, including burning sensations, dryness, and tearing in the eyes (14). Other CVS symptoms include asthenopia, blurry vision, eye strain, and slow focusing, which are linked to the fatigue of visual system components (14).

The psychosocial health issues brought by the excessive use of electronic devices are also beginning to receive more attention. Evidence, most of which has been conducted in Western countries, has shown that the excessive device use is associated with sleep disruption, parental relationship problems, school performance problems, mental health problems, and daytime fatigue (2, 15–19).

Most children in the United States, United Kingdom, Singapore, China, Norway, Japan, and many other countries, exceed the 2-h daily screen time limit recommended by the American Academy of Pediatrics and the HKSAR Department of Health (7, 17, 18, 20–22). In the United Kingdom, over 60% of adolescents aged 15 spent more than 2 h watching TV per day (23). Similar figures were reported in a Norwegian study conducted in 2013 (20). Granich and colleagues found that up to 87% of Australian children used screen-based media for over 2 h on a daily basis (24). It has also been reported that American children spent 6.43 h on screen-based media per day, on average (21). Notably, research has shown that symptoms which develop in childhood and adolescence due to extended device use predispose those individuals to a higher risk of musculoskeletal and visual system disorders in adulthood (6, 25).

Although knowledge about the negative impact of device use in youth is gradually increasing, there remains a great deal that is not yet known. For example, very few studies have examined the role of device use on psychosocial health; even fewer have compared children in primary school and adolescents in secondary school regarding the prevalence and severity of symptoms associated with electronic device use.

Given these considerations, the objectives of the current study were to increase our understanding of the nature and impact of device use in children and adolescents. To address these objectives, we sought to estimate: (1) the overall amount of electronic device use and symptom severity in samples of primary and secondary school students, and if they differed as a function of age/education level, and (2) the associations between device use and the prevalence and severity of a variety of symptoms and quality of life domains. We hypothesized that (1) more than 50% of the both study samples would report an average daily use greater than or equal to the recommended maximum of 2 h/day, (2) older participants would report more electronic device use and symptom severity than younger participants, and (3) more electronic device use would be associated with higher prevalence and severity of symptoms, and lower device-related psychosocial health and quality of life.

## Methods

### Study design

A cross-sectional study design was adopted. Data on the demographics, electronic device use of the adolescent participants, point prevalence and severity (expressed in terms of frequency and intensity) of musculoskeletal symptoms, visual symptoms, psychosocial health, and quality of life were measured and collected by questionnaire.

### Recruitment procedure and data collection

Study participants were recruited via the convenience sampling, from schools in Hong Kong that: (1) were registered under the Hong Kong Education Bureau, (2) offered Primary 5–6 or Secondary 1–4 education (i.e., grades 5–10), and (3) did not focus on serving children with special needs. The inclusion criteria for the student participants are being: (1) a student in grades 5–10, (2) able to read and write Chinese or English, and (3) able to complete an online or hard copy version of the study questionnaire without assistance.

Consent for their child’s and adolescent’s participation was obtained from the parents of the student participants who were <18 years old. Signed consent for all of the student participants was obtained before the distribution of the questionnaire. The data were collected from September 2019 to March 2020. A teacher in each school distributed a hard copy of the questionnaires or provided a link for participants to complete the questionnaire online, after explaining the purpose, process and ethical issues of the study. It took 10–15 min to complete the study measures as reported by the teachers.

A total of 1,152 students from one primary and two secondary schools were approached. One thousand and seventy-three of these students and their parents consented to participate in the study, and 1,058 students (response rate of 92%) completed the study questionnaires.

### Ethical consideration and confidentiality

The study was approved by the Human Subjects Ethics Sub-Committee of the Hong Kong Polytechnic University (Reference Number: HSEARS20180604002). The completed questionnaires were stored in a secured place or encrypted storage and were not used for any purpose other than the study.

### Measures

#### Demographic variables and intensity of device use

The questionnaire asked participants to provide information regarding their demographics (i.e., age, sex, and class year), and to indicate the average daily hours of electronic device use on school days and on holidays.

#### Musculoskeletal-related symptoms

Participants were also asked to indicate the presence, frequency, and intensity of four musculoskeletal-related symptoms during and/or after using electronic devices (Supplementary material). Presence and frequency were assessed using a 4-point Likert scale (0 = “Never: The symptom/condition does not occur at all”; 1 = “Occasionally: Sporadic episodes or at most 1 time/week”; 2 = “Frequent: 2–3 times/weeks”; 3 = “Always: Almost every day/week”). For symptoms that were rated as being present, their intensity was assessed using a 3-point categorical scale (Mild = “You can feel the symptom, but it does not bother you”; Moderate = “The symptoms are bothering you and a break might be needed due to the symptom;” Severe = “The symptom bothers you so much that a treatment either by yourself or medical professionals is needed”). The four musculoskeletal symptoms assessed were pain or aches, stiffness, or tiredness in the (1) neck, (2) shoulder, and (3) back regions, and (4) feelings of pins and needles or numbness in the upper limbs.

#### Vision-related symptoms

The presence, frequency and intensity of nine visual symptoms were also assessed, using the same questions used to assess musculoskeletal symptoms. The symptoms chosen for assessment were based on those associated with CVS (14), and included eye dryness, eye burning, eye itching, tearing, eye redness, eye pain, blurred vision, difficulty focusing for near vision, and double vision.

#### Psychosocial health related to device use

We also asked participants to indicate the presence and frequency with which: (1) they used an electronic device longer than originally intended, (2) others complained about the participant’s electronic device use, (3) they attended school, (4) they perceived a negative impact of electronic device use on school performance, (5) they experienced sleep disturbance, and (6) were emotionally upset when not using electronic devices.

#### Musculoskeletal discomfort index, eye discomfort index, and device-related psychosocial health index

The responses to the questions assessing symptoms or problems in each of the above three domains were used to compute scores representing: (1) a Musculoskeletal Discomfort Index (MDI), (2) an Eye Discomfort Index (EDI), and (3) a Device-Related Psychosocial Health Index (DRPHI). A respondent was classified as being symptomatic if they had a value of >0 on the respective index (i.e., reported at least one episode of at least one symptom), and asymptomatic if they answered “Never” in response to all of questions related to a symptom domain. The internal consistency (Cronbach’s alpha) of the MDI, EDI and DRPHI in the current sample were 0.90, 0.85, and 0.79, respectively, indicating adequate to excellent reliability.

### Quality of life

Questions assessing additional health and quality of life domains that could potentially be impacted by device use were also assessed, and included: (1) change in refractive error over the last 12 months, (2) days spent on exercise per week excluding PE lessons, (3) average daily sleep duration, (4) quality of relationship with family and frequency of having disagreement with family members in the past 12 months, and (5) academic performance.

### Data analysis

Descriptive statistics were computed for all study variables for descriptive purposes. Study hypothesis 1 was tested by examining the percentage of respondents across both samples who reported using an electronic device for more than 2 h/day. We then conducted a series of Mann–Whitney tests to test the second study hypothesis; that is, to evaluate the differences in the time spent on electronic device during weekdays and weekend/holidays (the cumulative usage time/day collected in the questionnaire were in ordinal scale), musculoskeletal symptoms, visual symptoms, and psychosocial health between primary and secondary school students. We also computed Spearman’s rank coefficients between the participants’ education level (i.e., class year) and the average number of hours of device use. Finally, we tested the third study hypothesis by computing a series of Spearman’s rank correlation coefficients between the average number of hours of electronic device use and the study criterion variables (i.e., MDI, EDI and DRPHI scores, and responses to the five questions assessing quality of life domains). A value of p of less than 0.05 was considered to be statistically significant. Data were analyzed using IBM SPSS Statistics (Version 26.0, Armonk, NY, IBM Corp.).

## Results

### Demographics

Table 1 presents the descriptive information about the study sample. As can be seen the mean age (SD) of the entire study sample was 12.83 years (SD, 1.76; range, 9 to 17). Fifty-one percent were male and 50% were female (sums to >100% due to rounding error).

**Table 1:** Demographics including age and class years of the participants (*n* = 1,058).

**Age in years**	**Range**	**Mean (SD)**
	9–17	12.83 (1.76)
**Class year**		***n* (%)**
Primary 5		129 (12.19%)
Primary 6		119 (11.25%)
Secondary 1		237 (22.40%)
Secondary 2		191 (18.05%)
Secondary 3		192 (18.15%)
Secondary 4		190 (17.96%)

### Device use on school days, weekends and holidays

**Figure 1:**
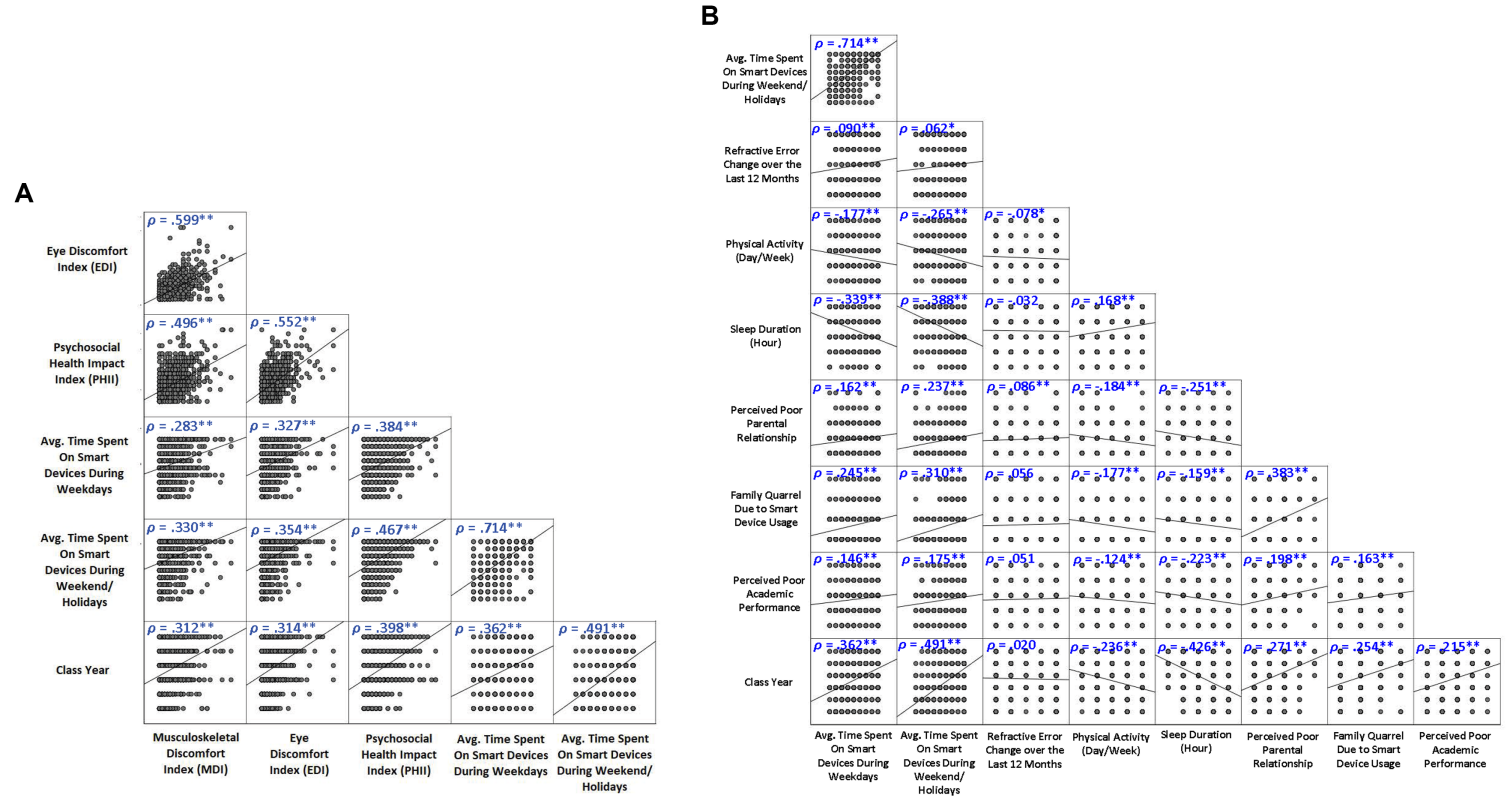
Spearman’s rank correlation between device use and **(A)** EDI, MDI and DRPHI, and **(B)** quality of life domains (^**^p < 0.01; ^*^p < 0.05).

Thirty-eight percent and 69% of the primary and secondary school participants used an electronic device ≥ 2 h/day. The average hours of electronic device use in secondary school students was significantly greater than in primary school students (*U* = 62,384, *p* < 0.001). Meanwhile, 44% and 90% of primary school and secondary students reported that they used an electronic device ≥ 2 h/day during weekends and holidays, respectively. The average duration of electronic device use was significantly higher in the secondary school group during weekends and holidays (*U* = 31,994, *p* < 0.001). Class year was positively and significantly associated with the average time spent on electronic devices during school days (*ρ* = 0.36, *p* < 0.001) and during weekends and school holidays (*ρ* = 0.49, *p* < 0.001; Figure 1).

### Prevalence and frequency of musculoskeletal and visual symptoms

**Figure 2:**
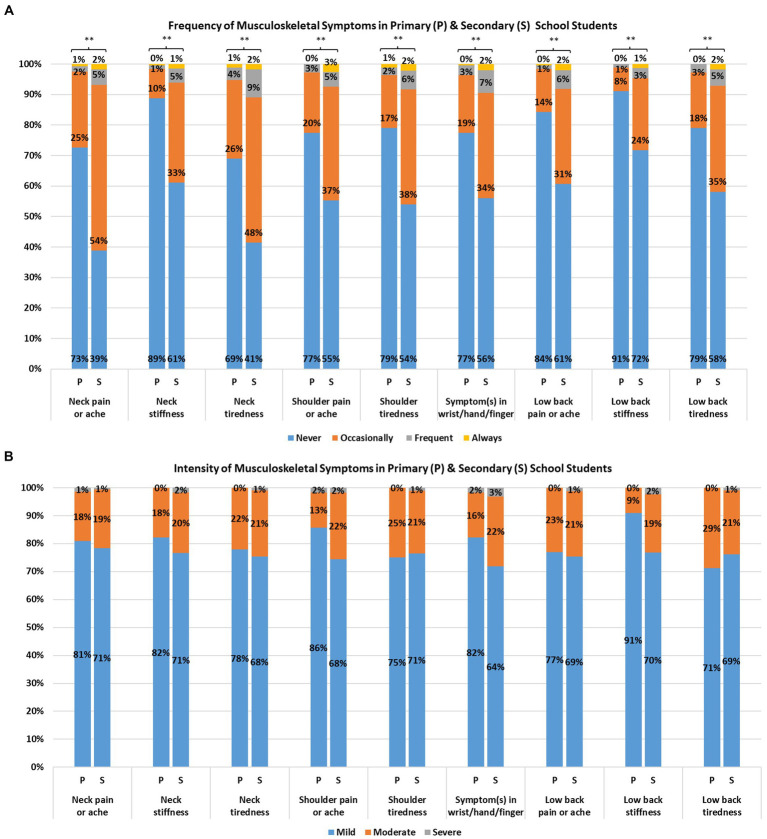
Self-reported frequency **(A)** and intensity **(B)** of musculoskeletal symptoms from primary and secondary school students (^**^p < 0.01).

**Figure 3:**
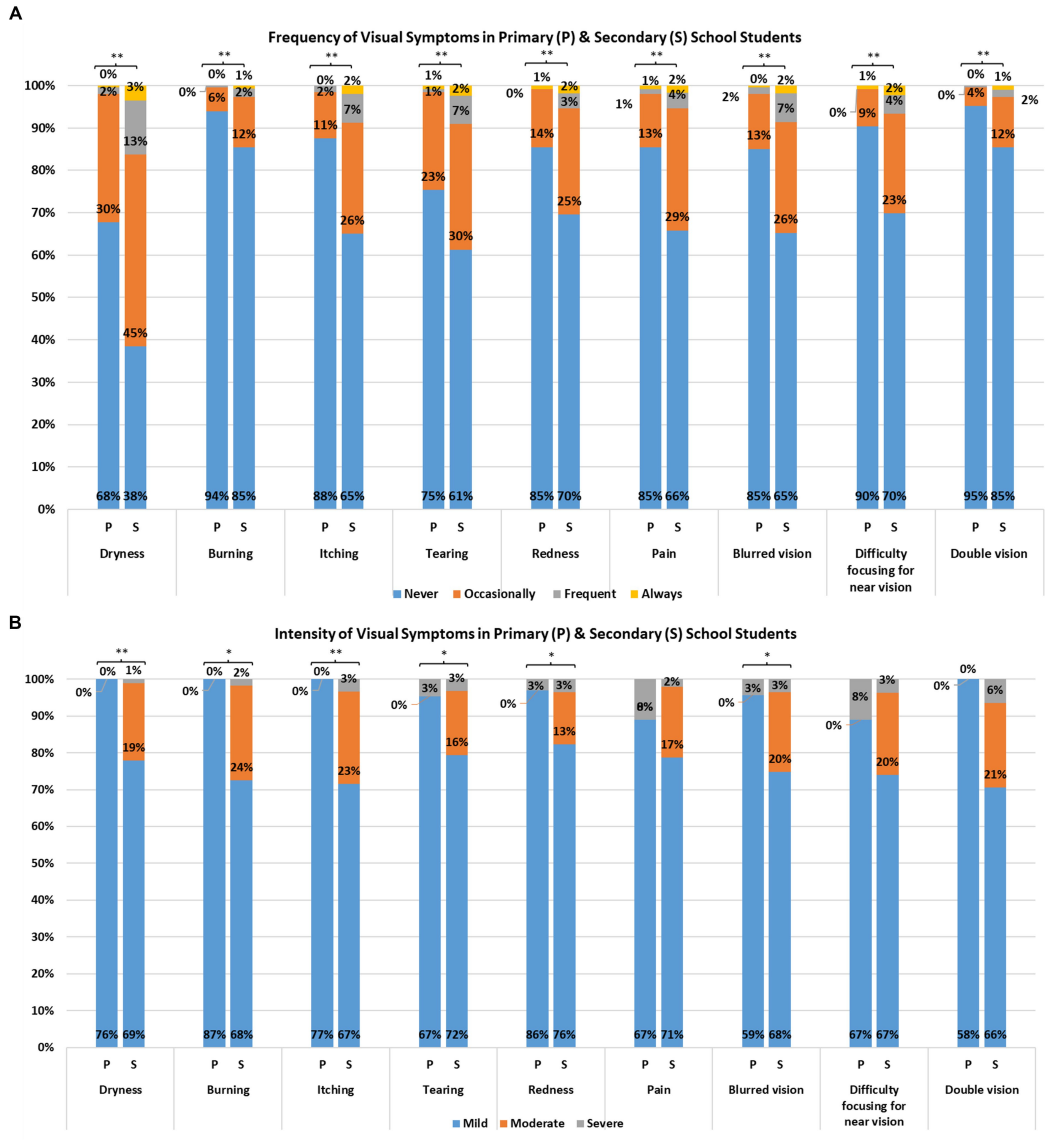
Self-reported frequency **(A)** and intensity **(B)** of visual symptoms from primary and secondary school students (^**^p < 0.01; ^*^p < 0.05).

Overall, 24% to 53% and 12% to 55% of participants endorsed having musculoskeletal and visual symptoms during and/or after electronic device use, respectively; the majority reported occasional symptoms (Figures 2, 3). More than half of the participants reported having at least occasional neck pain or aches (53%), neck tiredness (52%), and eye dryness (55%). For low back pain or ache, eye tearing, and eye dryness, the number of secondary school students endorsing the symptoms at least 2–3 times per week was around 5 to 8 times higher than primary school students.

The percentage of participants reporting musculoskeletal and visual symptoms was approximately 1.8 to 3.0 times higher in secondary school students than primary school students. The frequencies of all musculoskeletal and visual symptoms were significantly higher in secondary school participants (*U*’s = 65,838 to 91,109, *P*’s < 0.001; Figures 2, 3). The MDI and EDI were both significantly and positively correlated with the class year (*ρ*’s *= 0*.31 and 0.31; *P*’s < 0.001; Figure 1).

### Intensity of musculoskeletal and visual symptoms

In terms of intensity, 67% to 72% and 65% to 77% of all symptomatic respondents experienced mild musculoskeletal and visual symptoms, respectively (Figures 2, 3). Eighteen percent to 22%, and 11% to 21% of participants reported moderate musculoskeletal and visual symptoms. The percentage dropped to 1% to 3% and 1% to 5% for severe musculoskeletal and visual symptoms. The proportion of secondary school students endorsing moderate visual symptoms (13% to 24%) was higher than that of primary school students (0%). Statistically significant differences between primary and secondary school students in intensity were found for eye dryness (*U* = 10,492, *p* < 0.001), eye burning (*U* = 514, *p* = 0.03), eye itching (*U* = 2,256, *p* = 0.002), tearing (*U* = 5,194, *p* = 0.02), eye redness (*U* = 3,089, *p* = 0.04) and blurred vision (U = 2,340, *p* = 0.03), but not in the remaining visual and musculoskeletal symptoms.

### Device-related psychosocial health

**Figure 4:**
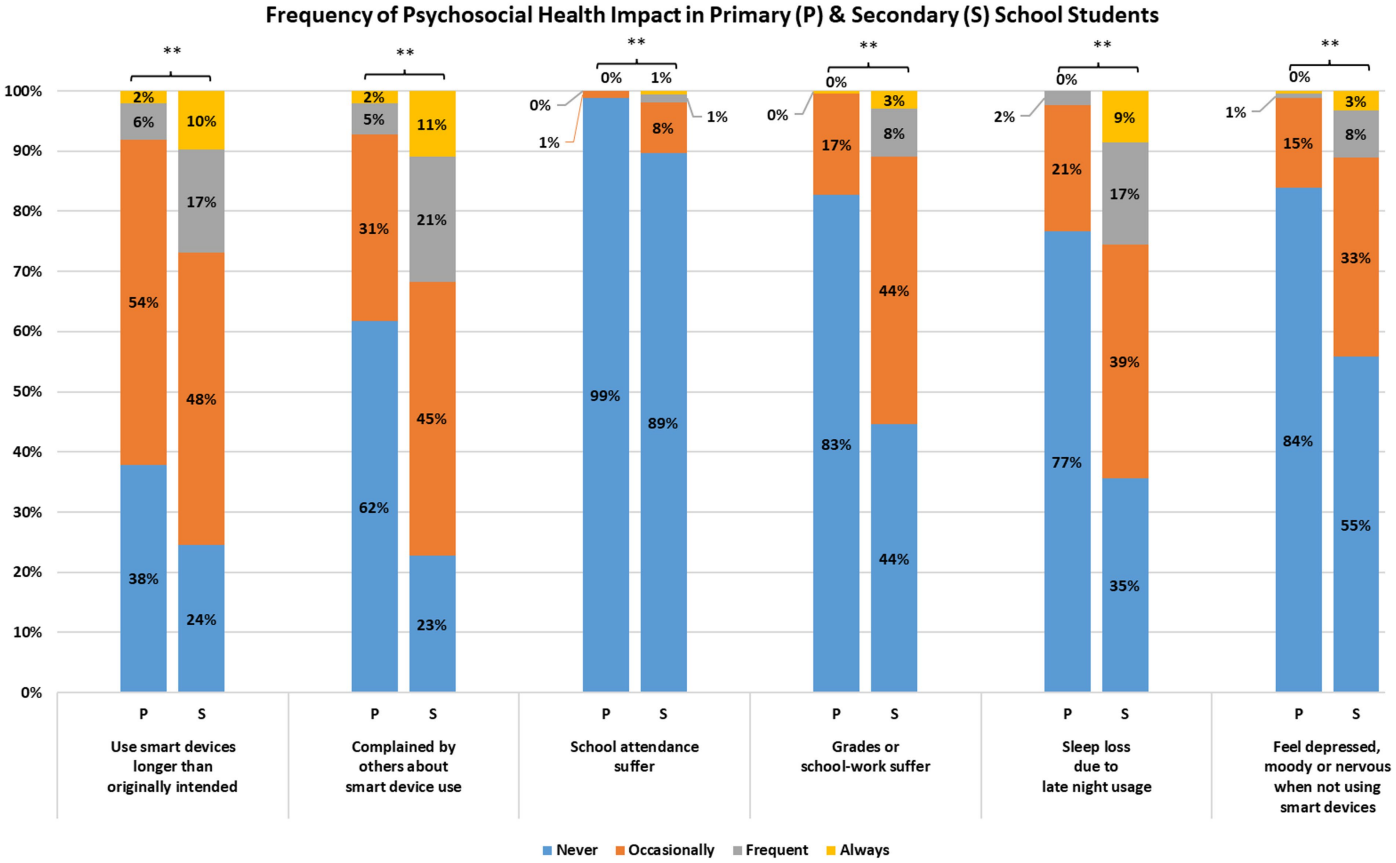
Self-reported frequency of psychosocial health issue from primary and secondary school students (^**^p < 0.01).

More than half of all respondents reported having at least some problems with electronic device use for 3 out of the 6 domains (Figure 4). This included reporting that (1) they used electronic devices longer than intended (72%), (2) others complained to them about the time spent on electronic devices (68%), and (3) sleep disruption due to late night usage of electronic devices (55%). The percentage of secondary school students endorsing these issues were generally higher (1.2 to 8.9 times) than that of primary school students. The frequencies of all 6 device-related issues were significantly higher in secondary school population (*U*’s = 54,182 to 90,839, *P*’s < 0.001; see Figure 4). A significant relationship was found between class year and DRPHI (*ρ* = 0.40, *p* < 0.001; see Figure 1). Finally, the rates of secondary school students endorsing device-related issues at least 2 to 3 times weekly (i.e., frequent or always) were about 3 to 9 times higher than that of primary school students. Significant relationships were found between the time spent on electronic device and reduced physical activity and sleep duration, and poorer family relationship and academic performance (Figure 1).

## Discussion

### Electronic device use among children and adolescents

The study findings are consistent with prior research reporting high use of electronic devices among children and adolescents (2, 7, 17, 18). Alarmingly, 18**%** and 36% of the study participants spent 4 h per day or more using electronic devices during school days and weekends/holidays, respectively, which is more than twice the time limit suggested by the American Academy of Pediatrics (22, 26). In addition, the findings showed that the secondary school students reported a greater use of electronic devices than did the primary school students. These findings highlight the critical importance of early intervention targeted at primary school or even earlier to minimize the development of electronic device habits in youth. While restraining the device use time on recreational purpose among the youth is crucial, it would not be practical to limit the use of electronic devices for learning since the computer-assisted instruction has become indispensable in the modern education. Musculoskeletal Symptoms.

The highest prevalence of musculoskeletal symptom reported was in the neck region, including neck pain or aches (53%) and neck tiredness (52%). Sustained cervical muscle contraction, flexed neck posture due to lower display placement, lack of postural breaks and poor ergonomic workstation setup are all potential mechanisms of musculoskeletal symptoms among device users (2, 7, 27–29). The higher severity of musculoskeletal symptoms in older participants could potentially be due to the extended use of electronic devices, increased access to smartphones, and greater academic burden that requires electronic device use for learning (3, 7, 18). One of the more straightforward ways to alleviate such physical impact might be to encourage frequent breaks in between classes (e.g., 5–10 min postural break for each hour of device use) or after-class activities to help vary the posture (8, 22). Adding more time for physical education, including activities that target musculoskeletal health, is another possible strategy that could be encouraged by teachers. These additional interval breaks or physical education class can reduce the risk of having physical symptoms, without sacrificing academic performance (30, 31).

The present findings revealed that greater musculoskeletal symptoms were associated with higher class year and more device use. These findings are consistent with those from a study conducted by Toh and colleagues, who found that the odds of musculoskeletal symptoms increases 4 to 7% for every hour of daily smartphone use (7). The portability offered by handheld electronic devices is a double-edged sword that allows for multitasking on the one hand, but may also result in people using the device for longer than intended on the other.

### Visual symptoms

Similar to musculoskeletal symptoms, greater electronic device use was significantly associated with visual symptoms. One possible cause for the development and maintenance of these symptoms is the reduced blink rate and increased number of incomplete blinks that can occur with device use. Low blink rates increase corneal exposure to air, causing tear evaporation, and resulting in dry eyes and ocular irritation (2, 7, 13, 32). Also, constant accommodation is required when focusing on a screen, with a short viewing distance, especially when the screen is small as it is in a smartphone (2). This can result in eye strains, causing asthenopia. The blue light emitted from the screen is also thought to be damaging the cornea and retina, and contribute to eye fatigue (13, 14, 32).

The severity of visual symptoms was also significantly and positively associated with class year. One of the possible explanations of this finding is the longer duration of electronic device use by older children for different purposes (3, 7). These findings also point to the critical need to develop and then implement more effective strategies as school routines (e.g., 20–20-20 eye resting rule and adding 2 h/day of outdoor activities) for reducing screen use in children and adolescents, especially given the potential long-term negative consequences of eye problems that develop during childhood (4, 33).

#### Device-related psychosocial health

Our results clearly show that screen use had a negative association with device-related psychosocial health. The association between prolonged device use and negative relationships with parents was evident, leading to quarrels which can contribute adverse parent-children relationship (2, 22). The disagreement between parents and children might be further amplified by mood swings due to poor sleep quality, because of late night use of electronic device (34). The findings suggest the need for parental education and training in how they can effectively help their children limit device use, while maintaining positive interactions.

Parents could potentially help by providing greater structure for their children’s device use, by allowing device use when children meet specific goals, such as accomplishing household chores or achieving satisfactory grades. Moreover, parents could also act as a positive role model for their children in terms of healthy use of electronic devices.

Sleep deprivation was another key psychosocial health issue. The length of sleep was inversely related to the time spent on electronic devices; a finding consistent with a study conducted by Parent and colleagues (35). Exposure to video games prior to sleep and viewing a bright screen while engaging in tasks linked to emotional responding (e.g., gaming or social media), could increase an adolescent’s psychophysiological arousal, thus interfering with sleep (36–38). Furthermore, the blue light emitted by the screens on many devices can interfere with melatonin production and the circadian rhythm (16, 38). Insufficient sleep, which is detrimental to the adolescent’s growth and development, is also associated with fatigue and poor academic performance (16, 39). Again, these findings point to the need to educate parents regarding effective strategies they can use to help limit the negative effects of device use on their children’s sleep quality.

## Limitations

This study has a number of limitations that should be considered when interpreting the results. First, the study sample did not include senior secondary school students (Secondary 5 and 6), because they were busy preparing for the local public exam for university entry. Additional research with a large sample size that include senior secondary school students to be recruited through random sampling method would be needed to promote the generalizability of the findings. Second, given that the data are cross-sectional, it is not possible to test for and draw conclusions about causal associations among the study variables. However, it seems unlikely that potential musculoskeletal and visual problems use would have a causal impact on the higher device usage among the children and adolescents.

## Conclusion

Despite the study’s limitations, the findings provide new information regarding the frequency of device use in children and adolescents, as well as the associations between this use and age, musculoskeletal problems, eye problems, and psychosocial health. It would appear that the traditional parenting approach to simply limit children’s access to electronic devices is not practical (3, 22). Additional efforts to provide children, adolescents, parents, and teachers with education about the healthy use of electronic devices (e.g., ergonomics, interval postural and visual breaks, physical activity) appears needed. Early intervention may be necessary to target the prolonged and improper use of electronic device among children at early age, to prevent the long-term health consequences, particularly when computer-assisted learning has become increasingly popular and common. This can be achieved by implementing the health education and screening, and large-scale longitudinal studies with the collaborative effort between various stakeholders, which include the youth, their parents and their schools as well as the public health policies to be set by the government.

## Data availability statement

The original contributions presented in the study are included in the article/Supplementary material, further inquiries can be directed to the corresponding author.

## Ethics statement

The studies involving human participants were reviewed and approved by Institutional Research Board, Hong Kong Polytechnic University. Written informed consent to participate in this study was provided by the participants’ legal guardian/next of kin.

## Author contributions

ST, GC, AL, AS, PP, K-CY, JC, and MJ: conception or design of the work, acquisition, analysis, or interpretation of data for the work, drafting and revising of the work, and approval of publication of the content. All authors contributed to the article and approved the submitted version.

## Funding

This research was supported by Health and Medical Research Fund, Health Bureau of Hong Kong (project number.: 02180348).

## Conflict of interest

K-CY was employed by Hong Kong Evangelical Church Social Service Limited, Hong Kong.

The remaining authors declare that the research was conducted in the absence of any commercial or financial relationships that could be construed as a potential conflict of interest.

## Publisher’s note

All claims expressed in this article are solely those of the authors and do not necessarily represent those of their affiliated organizations, or those of the publisher, the editors and the reviewers. Any product that may be evaluated in this article, or claim that may be made by its manufacturer, is not guaranteed or endorsed by the publisher.
